# Enhancing massed prolonged exposure with cannabidiol to improve posttraumatic stress disorder: Design and methodology of a pilot randomized clinical trial

**DOI:** 10.1016/j.conctc.2024.101270

**Published:** 2024-02-15

**Authors:** Casey L. Straud, John D. Roache, Brett C. Ginsburg, Rais M. Baig, Van L. King, Sarah Barron, Tabatha H. Blount, Stacey Young-McCaughan, Alan L. Peterson

**Affiliations:** a Department of Psychiatry and Behavioral Sciences, The University of Texas Health Science Center at San Antonio, San Antonio, TX, USA; b Department of Psychology, The University of Texas at San Antonio, San Antonio, TX, USA; c Polytrauma and Rehabilitation Clinic, South Texas Veterans Health Care System, San Antonio, TX, USA; d PTSD Clinic, South Texas Veterans Health Care System, San Antonio, TX, USA

**Keywords:** Posttraumatic stress disorder, Cannabidiol, Prolonged exposure therapy, Trauma-focused therapy

## Abstract

**Background:**

The impact of posttraumatic stress disorder (PTSD) is substantial and often results in pervasive functional impairments. Although evidence-based treatments for PTSD are established, there remains room for improvement as many individuals continue to meet diagnostic criteria even after successful treatment completion. Cannabidiol (CBD) has attracted considerable attention based on its potential to treat a myriad of health conditions. CBD may decrease anxiety and facilitate extinction learning processes, two critical targets of trauma-focused psychotherapies. We present the design and methods for a pilot randomized clinical trial to examine the combination of CBD and prolonged exposure for PTSD.

**Methods:**

Participants (*n =* 24) will be randomized to CBD or placebo for 18 days delivered in combination with ten daily prolonged exposure sessions over two weeks. The study medication will be Epidiolex® (250 mg BID). The PTSD Checklist for DSM-5 will be the primary outcome to assess PTSD severity at baseline, during treatment, and at 1-month follow-up. Blood, saliva, and heart rate will be collected during treatment to assess intervention effects on biological outcomes related to PTSD and the endocannabinoid system.

**Results:**

Consistent with the purpose of a pilot, our goals are to evaluate the feasibility of study procedures, safety of the intervention, and the preliminary effect of CBD to inform a larger trial. Descriptive and inferential statistics will be used to address study aims.

**Conclusion:**

Findings will inform decision making on combining CBD with behavioral interventions for PTSD to enhance outcomes and mitigate the morbidity of this debilitating condition.

## Highlights

• Novel treatments for PTSD are critical given that many do not achieve remission after treatment.
• CBD may decrease hyperarousal and enhance extinction learning.
• The design and methods for a pilot RCT to examine a novel combination intervention are presented.
• Findings will inform planning for a larger RCT and an alternative treatment option for PTSD.

## 1 Introduction

Approximately 70% of the U.S. population will experience a traumatic event in their lifetime, and 8% of those individuals will go on to develop posttraumatic stress disorder (PTSD) [1]. Symptoms of PTSD can include intrusive memories, nightmares, anxiety, hypervigilance, avoidance of trauma reminders, and negative mood and cognitions. The impact of PTSD is substantial and often results in pervasive functional problems with health, relationships, and work. Given the prevalence, chronicity, and debilitating nature of PTSD, the need for more effective PTSD treatments is critical.

Evidence-based treatments for PTSD exist and are well-established. Trauma-focused cognitive behavioral therapies are consistently recommended as first-line interventions for PTSD [2]. Prolonged Exposure (PE) is one of the most widely used and empirically supported trauma-focused therapies, and has been validated across various populations and trauma types as a first-line intervention for PTSD [[3], [4], [5], [6], [7], [8]]. The standard PE protocol involves approximately 8–15, 90-min weekly sessions and includes homework exercises between treatment sessions. PE aims to reduce trauma-related distress and PTSD symptomatology through extinction learning interventions (i.e., repeated exposure) to internal (e.g., trauma memory) and external (e.g., environmental stimuli) trauma cues under objectively safe conditions. Despite evidence supporting trauma-focused therapies such as PE, there remains room for improvement given dropout rates (36%) and the large proportion (∼50%) of patients that do not achieve PTSD remission following treatment [9,10].

Although trauma-focused therapies have demonstrated the greatest efficacy, pharmacotherapy remains the most commonly used approach to treat PTSD [11]. The only pharmacological interventions for PTSD approved by the Food and Drug Administration (FDA) are the selective serotonin reuptake inhibitors (SSRIs) fluoxetine and sertraline. Unfortunately, the ability of these pharmacological approaches to reduce PTSD symptoms is limited [12,13]. These drugs can also have considerable adverse effects profiles. Based upon expert clinical practice guidelines, no medications have been determined to be a first-line treatment option for PTSD over trauma-focused psychotherapies [14]. Limitations on the benefits of any of these pharmacotherapies have prompted the use of polypharmacy, whereby patients are prescribed a combination of medications with little empirical guidance on either the benefits or adverse reactions of this approach [15]. Novel, alternative pharmacotherapies that more effectively target trauma symptoms and address the same critical intervention processes known to be effective in trauma-focused therapies for PTSD are critical, especially if combined with psychotherapy to achieve enhanced therapeutic benefit.

### 1.1 Cannabinoids and PTSD

Cannabinoids have attracted considerable attention in recent years based on their purported ability to treat a myriad of physical and psychiatric conditions via the endocannabinoid system (ECS) [16]. The ECS is comprised of two primary receptors (CB1 and CB2), two endogenous neurotransmitters (anandamide [AEA] and 2-arachidonylglycerol [2-AG]), and several metabolic enzymes (fatty acid amide hydrolase and monoacylglycerol lipase). The ECS plays a critical role in modulating anxiety and pain, with emerging evidence indicating a potential role in PTSD treatment [17]. Existing research has found a negative relationship between activity in the ECS and trauma, such that the ECS activity may modulate the stress response and exposure to psychological trauma may lead to long-term reductions in ECS activity [18,19]. Novel pharmacological approaches targeting the ECS have promising potential to improve mental and physical health, all of which are important to individuals struggling with the sequalae of trauma exposure.

Among the possible cannabinoid therapeutics, whole plant cannabis (marijuana) and several other constituents from the cannabis plant are of great interest. Delta-9-tetrahydrocannabinol (THC) and cannabidiol (CBD) are two of the primary constituents found in cannabis and interact with the ECS in different ways. THC acts as an agonist with high affinity for ECS CB1 and CB2 receptors [17]. THC is considered the primary cannabinoid found in the cannabis plant, and is responsible for its intoxicating effects and abuse potential. On the other hand, CBD has weak affinity for ECS receptors and the mechanism of action is more complex, involving multiple systems and indirect effects [20]. In contrast to THC, CBD does not cause intoxication and may offer the benefits associated with cannabis [17]. CBD is legal in most of the U.S. and is no longer a federally controlled substance when extracted from low THC-containing hemp (<0.3 % by weight). CBD is well tolerated, even in large doses (1500 mg) with mild adverse side effects (e.g., nausea, headache, drowsiness) [21].

Preclinical models have shown that CBD can reduce cardiovascular and anxiogenic effects of stress [22,23]. CBD has also been found to decrease retrieval and acquisition of fear memories, block reconsolidation of the trauma memory, and facilitate the extinction learning process in animal models [[18], [19], [24], [25], [26]]. Consistent with preclinical research, early human studies have found that CBD facilitated extinction learning and decreased cue-based fear response in healthy subjects [27]. A case report demonstrated that daily use of CBD was associated with reduced anxiety and improved sleep in a treatment resistant PTSD patient [28]. In a case series study, CBD was related to PTSD severity reductions and was well tolerated by the sample (no one discontinued treatment due to adverse effects of the medication) [29]. However, the first randomized clinical trial (RCT) of cannabis for PTSD suggested that monotherapy with CBD is unlikely to lead to significant PTSD reductions [30]. Indeed, CBD monotherapy may provide short-term symptom relief, but may not be effective in maintaining long-term PTSD symptom reductions [31]. Collectively, early findings still suggest possible benefit of CBD treatment for PTSD for several reasons. First, CBD may reduce PTSD pathology associated with the stress response. Second, CBD is a therapeutic constituent of cannabis but is legal, well tolerated, and without the intoxicating effects associated with THC. Third, CBD may reduce symptomatic factors (i.e., anxiety) associated with trauma-focused psychotherapy refusal and dropout. Finally, the greatest potential for CBD may be as a facilitator of engagement in trauma-focused therapy based on its ability to enhance extinction learning.

### 1.2 Research aims and objectives

In this manuscript, we present the design, methods, and analytic plan for a pilot, placebo controlled RCT to examine a novel combination intervention of CBD and PE and to review the relationship between the ECS, CBD, and PTSD. Our specific aims are to: 1) examine the safety, feasibility, and benefits of CBD among individuals receiving PE for PTSD; 2) evaluate biological outcomes associated with the ECS and stress response; and 3) explore the association between changes in related biological outcomes and PTSD severity following treatment.

## 2 Materials and methods

### 2.1 Study design

**Fig. 1:**
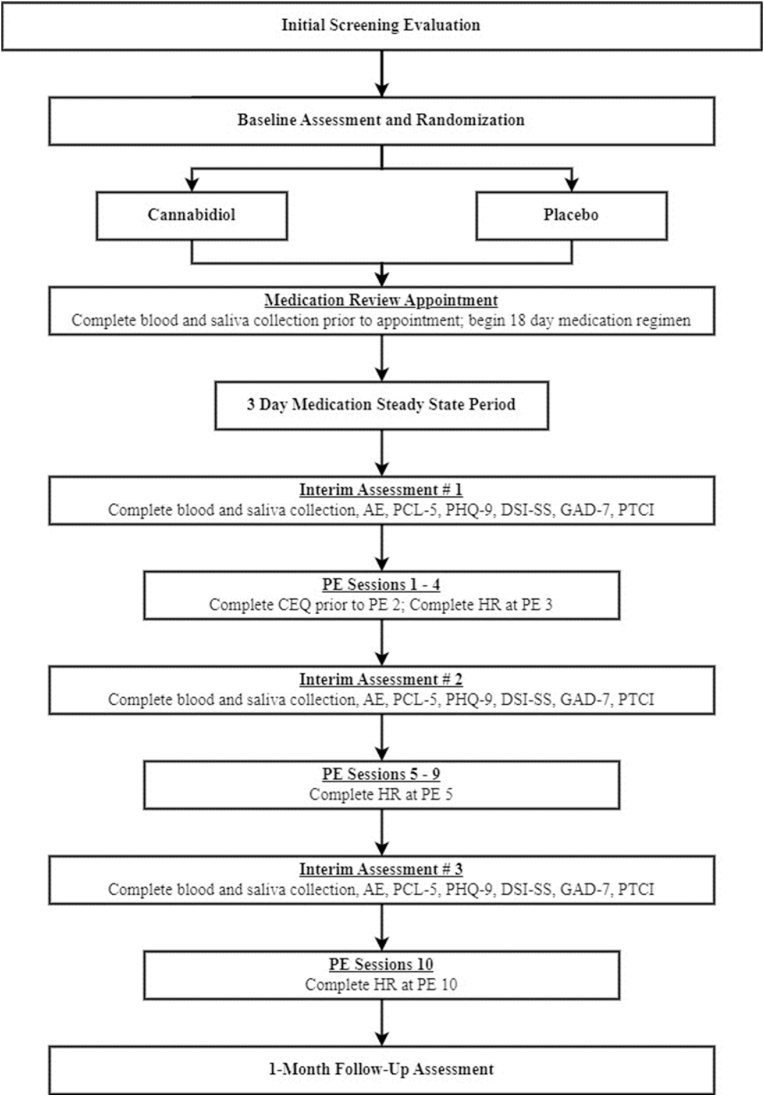
Study Overview Flow Chart. AE, Adverse Event Monitoring; PCL-5, Posttraumatic Stress Disorder Checklist for DSM-5; PHQ-9, Patient Health Questionnaire-9; DSI-SS, Depressive Symptoms Index – Suicidality Subscale; GAD-7, Generalized Anxiety Disorder-7; PTCI, Posttraumatic Cognitions PE, prolonged exposure; CEQ, Credibility and Expectancy Questionnaire; HR, Heart Rate.

The overall study design is presented in Fig. 1. This study is a pilot, two-arm, double-blind, placebo controlled, fixed dose RCT to evaluate CBD combined with PE for the treatment of PTSD. Participants will be 24 individuals with PTSD randomized to CBD (*n* = 12) or placebo (*n* = 12) for 18 days delivered in combination with ten, 90-min sessions of massed, daily PE over two weeks as a standard of care. Research has found that massed delivery of PE reduces attrition while maintaining equally efficacious outcomes [6]. Following treatment, regardless of the extent of participation, individuals will be asked to complete a follow-up assessment that will occur approximately 1-month after the last day of treatment.

### 2.2 Randomization and blinding

A permuted block randomization design stratified by PTSD severity (median PCL-5 score of 51) and population type (military or civilian) will be used to determine group assignment. The median PCL-5 score used herein was derived from two RCTs recently completed by the South Texas Research Organizational Network Guiding Studies on Trauma and Resilience (STRONG STAR) Consortium and informed by known differences in treatment response between military and civilian samples [8,32,33]. One investigator (JDR) with no study participant contact will develop the permuted blocked randomization sequences for each of four stratification groups, such that two CBD and two placebo randomizations will occur within each block of four randomizations. All other study team members will be blinded to randomization sequence. For each eligible participant, the compounding pharmacy will be provided with the participant study number and stratification information so that the pharmacist can assign the patient to the next randomization sequence. The pharmacist will prepare CBD or placebo solutions to be dispensed in blinded containers identified with a participant name and identification number and labeled as “CBD/Placebo for investigational use only.”

### 2.3 Participant recruitment & screening

All participants will be recruited through the San Antonio community, hospitals, and clinics through provider referrals, recruitment events, and flyers. Study information will also be posted on the STRONG STAR clinic website and social media by our communications team for self-referred participants. Study personnel will initially conduct a brief telephone interview to prescreen each individual. Participants who appear eligible after telephone prescreening will be invited to the University of Texas Health Science Center at San Antonio STRONG STAR clinic to provide written informed consent and undergo more rigorous assessment for study eligibility. Following consent, a baseline assessment will take place to determine participant eligibility. The baseline screening will include a battery of self-report questionnaires, clinical interviews, and biological screening tests.

### 2.4 Inclusion criteria

Detailed inclusion and exclusion criteria are provided in Table 1. The inclusion criteria for this study will be as follows: 1) individuals between 18 and 65 years old*;* 2) able to write, read and speak English; 3) PTSD diagnosis as assessed by the Clinician-Administered Posttraumatic Stress Scale (CAPS-5) [34]; 4) stable medication regimen for at least four weeks prior to the onset of study participation; and 5) able to participate in daily PE for a 2-week period.

**Table 1:** Inclusion and exclusion criteria.

Inclusion Criteria:
1) Individuals between 18 and 65 years old. 2) PTSD diagnosis as assessed by the CAPS-5 3) Stable medication regimen for at least four weeks prior to the onset of study participation 4) Able to participate in daily PE for a 2-week period. 5) Able to write, read and speak English.

Exclusion Criteria
1) Currently using opiates, cocaine, methamphetamines, or cannabis as measured by a positive urine drug screen. 2) Current alcohol abuse severity warranting designation as the primary disorder and requiring immediate intervention based on the QDS and clinical judgment. 3) History of drug abuse in the past year where study participation would be contraindicated based on the NIDA-Q and clinical judgement. 4) ALT/AST enzyme levels 3 times the normal limits via liver function test completed within the last 90 days. 5) Ongoing illness or physical health problems that puts participants at increased risk of adverse effects in the clinician's judgement. 6) History of significant hypersensitivity to cannabinoids or sesame seed oil. 7) Concurrently engaged in another trauma-related therapy. 8) Psychosis, mania, or suicide risk requiring immediate stabilization or hospitalization. 9) Currently pregnant (urine screen) or breast feeding. 10) Regular use of concomitant medications because of interactions with CBD metabolism: a) anticonvulsants carbamazepine and phenytoin. b) atypical antipsychotics olanzapine, quetiapine, and risperidone. c) any barbiturate or chronic, daily use of benzodiazepines [intermittent or as needed dose permitted]. d) regular hypnotic use with zaleplon, zolpidem, eszopiclone, ramelteon, and suvorexant. e) st. john's wort. f) oral use of glucocorticoids or unstable use of platelet inhibitors clopidogrel and warfarin. g) antiretrovirals ritonavir and indinavir or oral use of antifungals itraconazole and ketoconazole. h) antibiotics clarithromycin, erythromycin, and rifampin. i) chronic, daily use of opioids.

*Notes.* CAPS-5, Clinician-Administered Posttraumatic Stress Scale for DSM-5; PE, Prolonged Exposure; NIDA-Q, National Institute of Drug Abuse Quick Screen; QDS, Quick Drinking Screen; ALT, Alanine transaminase; AST, aspartate transaminase; CBD, Cannabidiol.

### 2.5 Exclusion criteria

The exclusion criteria for this study will be based on 10 determinants as follows: 1) currently using opiates, cocaine, methamphetamines, or cannabis as determined by a positive urine drug screen; 2) current alcohol abuse severity warranting designation as the primary disorder and requiring immediate intervention; 3) history of drug abuse in the past year where study participation would be contraindicated; 4) Alanine transaminase or aspartate transaminase enzyme levels 3 times the normal limits; 5) ongoing illness or physical health problems where study participation would put the individual at increased risk of adverse effects; 6) history of significant hypersensitivity to cannabinoids or sesame seed oil; 7) concurrently engaged in another trauma-related therapy; 8) psychosis, mania, or suicide risk requiring immediate stabilization or hospitalization; 9) currently pregnant or breast feeding; 10) regular use of prohibited concomitant medications.

### 2.6 Cannabidiol and placebo conditions

Participants will be randomized to 18 days of CBD (Epidiolex®) or placebo taken twice daily in the morning and evening after meals with high fat content as CBD has high lipophilicity. Epidiolex is an FDA approved, non-controlled prescription medication indicated for the treatment of seizure disorders [35]. Epidiolex comes in a 100 mL bottle and contains 100 mg of CBD per mL of strawberry-flavored liquid solution that is taken using an oral syringe. Dosing regimen will be a fixed dose of 2.5 mL (250 mg) taken twice daily for a total of 18 consecutive days consistent with starting recommended dose. Steady state concentration of CBD in the body has been shown to occur in 2–3 days. Accordingly, medication will be given for 3 days prior to beginning PE therapy on Day 4 [21]. The pharmacist will compound a matching placebo using only the inactive ingredients of Epidiolex to maintain the study blind. Medications will be dispensed in amber-colored bottles containing 92 mL of solution, a slight excess supply to account for treatment extension due to missed visits or trivial dosing errors. Blinded study medication will be stored in a locked cabinet in a temperature controlled, windowless room until the participant arrives at the clinic.

Participants will attend a medication review appointment (day 1 of treatment) and be provided information on proper use and storage of the study drug. A trained study team member will address any questions from the participant, and the participant will orally consume their first dose with a trained team member present to provide feedback. Participants will return to the clinic on day 4 to allow for an adequate steady state dose to be achieved prior to beginning PE.

### 2.7 Massed prolonged exposure

PE utilizes exposure-based interventions to target psychological mechanisms (i.e., experiential and behavioral avoidance; maladaptive cognitions) that maintain trauma-related symptoms and includes four primary treatment components: 1) psychoeducation on common reactions to trauma; 2) relaxation training; 3) in vivo exposure; and 4) imaginal exposure. In vivo exposure involves approaching avoided situations, people, places, and/or objects that are objectively safe (e.g., a crowded environment). Imaginal exposure involves repeatedly and systematically revisiting the trauma memory and related thoughts and feelings. Participants will be asked to complete homework, including reviewing treatment materials, listening to recordings of their imaginal exposure, and completing in vivo exposures. For this study, PE will be delivered daily on weekdays (not including holidays) over two weeks based on prior research demonstrating massed format decreases attrition while maintaining efficacy [6]. Each participant will receive ten, 90-min PE sessions over two weeks. The study team will work with participants to receive a release from work while participating in the treatment or offer HIPAA compliant virtual psychology appointments, as needed. All study therapists were trained to fidelity to conduct PE.

### 2.8 Outcome measures

Participants will complete a battery of self-reports, clinical interviews, and biological measures for screening purposes and outcome assessment. A strength of this study includes repeated assessment of subjective psychological symptoms and biological PTSD and ECS outcomes to examine CBD effects. Participants will complete interview and self-report measures conducted by condition-blind evaluators at baseline and 1-month follow-up, with select measures occurring at interim assessments (IA) 1, 2, and 3 (corresponding to PE sessions 1, 5, and 10). Blood and saliva collection will also occur at IA 1, 2, and 3 and will be carried out by trained team members. Heart rate will be collected during the imaginal exposure at sessions 3, 5, and 10 (final session). The primary outcome endpoints to satisfy study aims will be change from baseline to final PE session (benefit) and change from final PE session to 1-month follow-up (maintenance). A list of assessments and administration timing is provided in Table 2.

**Table 2:** Assessment schedule.

SCREENING MEASURES	BL	IA1	S2	S3	IA2	IA3	1MFU
Clinician-Administered PTSD Scale for DSM-5	X						X
STRONG STAR Health Questionnaire	X						X
Prior and Concomitant Medication Interview	X						X
Quick Drinking Screen	X						X
National Institute of Drug Abuse-Quick Screen	X						
Self-Injurious Thoughts and Behaviors Interview	X						X
Life Events Checklist	X						
Deployment Risk and Resilience Inventory-2	X						
Liver Function Test	X						
Urine Screen for THC and exclusionary substances	X						
Pregnancy Urinalysis (for female participants)	X						

PRIMARY OUTCOMES	BL	IA1	S2	S3	IA2	IA3	1MFU
Credibility and Expectancy for CBD/PE Questionnaire			X				X
Adverse Event Monitoring		X			X	X	X
PTSD Checklist for the DSM-5	X	X			X	X	X
Blood Draw and Saliva		X			X	X	
Heart Rate				X	X	X	

SECONDARY OUTCOMES	BL	IA1	S2	S3	IA2	IA3	1MFU
Depressive Symptom Index – Suicidality Subscale	X	X			X	X	X
Generalized Anxiety Disorder Screen	X	X			X	X	X
Patient Health Questionnaire-9	X	X			X	X	X
Posttraumatic Cognitions Inventory	X	X			X	X	X
Insomnia Severity Index	X						X
Brief Inventory of Psychosocial Functioning	X						X
Veterans RAND 12 Item Short Form	X						X

*Notes.* BL, baseline; S3, Session 3; IA, Interim Assessment (1, S1; 2, S5, 3, S10); 1MFU.

1-month follow-up.

#### 2.8.1 Interview and self-report measures

This study will examine the feasibility, safety, and preliminary intervention response of CBD with PE for PTSD. Feasibility (Aim 1a) will be based on screening, enrollment, initiation, and treatment adherence (e.g., sessions attended, medication compliance [self-report and blood levels], and session deviation log) numbers tracked by the study team. Adverse event (AE) monitoring will assess safety and will be tracked at each PE session and 1-month follow-up (Aim 1b). At AE assessment time points, participants will be queried on the severity of the event, the impact of the event on their overall functioning, and whether the event was associated with the study. The PTSD Checklist for DSM-5 (PCL-5) [36]. will be the primary outcome measure to assess PTSD severity reductions and will be administered at baseline, IA 1, IA 2, IA 3, and at the 1-month follow-up (Aim 1c). The CAPS-5 will also be administered to assess PTSD severity and to determine PTSD diagnosis at baseline and remission following treatment at the 1-month follow-up.

#### 2.8.2 Biological measures

Saliva and blood specimens will be collected prior to the pretreatment medication review, and at IA 1, 2, and 3 to assess levels of cortisol (saliva) and the endocannabinoids AEA and 2-AG (blood). Participants will complete biospecimens in a fasted state, having not taken their morning dose to capture a trough level medication concentration. Approximately 5 mL of blood will be collected by venous puncture, using a 5 mL EDTA blood collection tube immediately prior to the session. Saliva will be collected using a Salivette tube. Participants will be instructed on how to collect saliva samples immediately upon waking and asked to bring it to the scheduled appointment that day. Biospecimens will be chilled and transported to the University of Texas Health Science Center at San Antonio, Biological Psychiatry Analytical Laboratory for processing and storage until the time of analysis. Heart rate will be monitored using a Fitbit watch placed on the right wrist to obtain an objective arousal measure.

### 2.9 Data analysis plan

Consistent with the purpose of a pilot, this RCT is not powered for formal hypotheses testing [37]. Our primary interests are to evaluate the feasibility of study procedures, safety of the intervention, and the preliminary effect of CBD on PTSD to inform a larger trial. We will perform statistical analyses appropriate for an adequately powered study to identify data analysis issues germane to future planning (e.g., data management and scoring, missing data, data distributions, outliers, trends over time, covariance structures). Statistical analyses will be intent-to-treat. We will complete descriptive statistics to address the feasibility and safety aims. Inferential statistics will be used to explore the intervention effect on PTSD and biological markers. We will calculate Hedges' *g* with 95% confidence intervals to further describe the nature of effects. Hedges' *g* is recommended for small samples and can be interpreted using the same conventional recommendations as Cohen's *d*. Participants who discontinue treatment will be asked their reasons at the point of drop-out.

## 3 Discussion

This paper provides an overview of the research methodology and rationale for a pilot, placebo controlled RCT to examine the feasibility, safety, and effect of CBD for PTSD in a sample of individuals receiving PE as a standard of care. The interest and use of cannabinoid products for the treatment of various physical and mental health conditions is growing rapidly in the U.S. and among individuals with PTSD [38,39] There is minimal information available regarding the potential benefits and harms of cannabis products and many individuals resort to using cannabis products including marijuana to cope with symptoms when they fail to respond to available treatments [40]. More research is needed to fully understand the benefits of CBD for PTSD and other psychological disorders. Currently, there are no published RCTs investigating the effects of CBD to reduce PTSD symptoms when delivered in combination with trauma-focused psychotherapy, though limited existing research suggests that CBD could enhance the benefits of trauma-focused therapies [31,41]. This study will be one of the first RCTs to evaluate the combination of CBD with an evidence-based, trauma-focused psychotherapy for PTSD. To our knowledge, there is only one other registered clinical trial investigating CBD and trauma-focused psychotherapy and its results have not yet been published (NCT03518801). Both trials aim to combine CBD with PE; however, in the other trial PE is delivered in standard format (weekly) over 16 weeks vs. the 2-week massed (daily) format proposed in the current paper. Furthermore, the other trial does not list any biological outcome aims to examine the association between PTSD and CBD (e.g., heart rate, cortisol, and the endocannabinoids AEA and 2-AG).

Overall, the use and demand for cannabinoid for the treatment of various physical and mental health conditions is rapidly growing. More research is needed to fully understand the benefits of CBD for PTSD and other psychological disorders. Currently, there are no published RCTs investigating the effects of CBD to reduce PTSD delivered in combination with trauma-focused psychotherapy, though limited existing research suggests that CBD could enhance the outcomes of brief trauma-focused therapies. If the current project successfully demonstrates the feasibility and acceptability of CBD and PE for PTSD, it will provide a compelling rationale for a well-powered RCT to examine the efficacy of CBD combined with trauma-focused psychotherapy. The combined intervention could offer a novel, alternative treatment that may prove more effective and engaging than currently available PTSD interventions. Thus, mitigating the morbidity of this chronic and debilitating condition.

## Funding statement

This work was supported by a research grant to Casey L. Straud by the 10.13039/100006108National Center for Advancing Translational Sciences, National Institutes of Health, through Grant KL2 TR002646. The content is solely the responsibility of the authors and does not necessarily represent the official views of the NIH.

## Clinical trials registration

NCT05132699.

## CRediT authorship contribution statement

**Casey L. Straud:** Writing – review & editing, Writing – original draft, Project administration, Methodology, Investigation, Funding acquisition, Conceptualization. **John D. Roache:** Writing – review & editing, Supervision, Project administration, Funding acquisition, Conceptualization. **Brett C. Ginsburg:** Writing – review & editing, Writing – original draft, Supervision, Project administration, Formal analysis, Conceptualization. **Rais M. Baig:** Writing – review & editing, Supervision, Resources, Investigation, Conceptualization. **Van L. King:** Writing – review & editing, Supervision, Investigation. **Sarah Barron:** Writing – review & editing, Resources. **Tabatha H. Blount:** Writing – review & editing, Supervision, Investigation. **Stacey Young-McCaughan:** Writing – review & editing, Project administration, Investigation, Conceptualization. **Alan L. Peterson:** Writing – review & editing, Supervision, Resources, Project administration, Funding acquisition, Conceptualization.

## Declaration of competing interest

The authors have no conflicts of interest to report.

## Data Availability

Data will be made available on request.
